# Cystic Fibrosis of the Pancreas: The Role of CFTR Channel in the Regulation of Intracellular Ca^2+^ Signaling and Mitochondrial Function in the Exocrine Pancreas

**DOI:** 10.3389/fphys.2018.01585

**Published:** 2018-12-20

**Authors:** Tamara Madácsy, Petra Pallagi, Jozsef Maleth

**Affiliations:** ^1^First Department of Medicine, University of Szeged, Szeged, Hungary; ^2^HAS-USZ Momentum Epithel Cell Signalling and Secretion Research Group, Szeged, Hungary; ^3^Department of Public Health, University of Szeged, Szeged, Hungary

**Keywords:** cystic fibrosis transmembrane conductance regulator, epithelial cells, Ca signaling, mitochondrial damage, cystic fibrosis, exocrine pancreas

## Abstract

Cystic fibrosis (CF) is the most common genetic disorder that causes a significant damage in secretory epithelial cells due to the defective ion flux across the cystic fibrosis transmembrane conductance regulator (CFTR) Cl^-^ channel. Pancreas is one of the organs most frequently damaged by the disease leading to pancreatic insufficiency, abdominal pain and an increased risk of acute pancreatitis in CF patients causing a significant decrease in the quality of life. CFTR plays a central role in the pancreatic ductal secretory functions by carrying Cl^-^ and HCO_3_^-^ ions across the apical membrane. Therefore pathophysiological studies in CF mostly focused on the effects of impaired ion secretion by pancreatic ductal epithelial cells leading to exocrine pancreatic damage. However, several studies indicated that CFTR has a central role in the regulation of intracellular signaling processes and is now more widely considered as a signaling hub in epithelial cells. In contrast, elevated intracellular Ca^2+^ level was observed in the lack of functional CFTR in different cell types including airway epithelial cells. In addition, impaired CFTR expression has been correlated with damaged mitochondrial function in epithelial cells. These alterations of intracellular signaling in CF are not well characterized in the exocrine pancreas yet. Therefore in this review we would like to summarize the complex role of CFTR in the exocrine pancreas with a special focus on the intracellular signaling and mitochondrial function.

## Cystic Fibrosis Transmembrane Conductance Regulator and Its Role in Pancreatic Physiology

Since its discovery in 1989 (Kerem et al., 1989; Riordan et al., 1989) cystic fibrosis transmembrane conductance regulator (CFTR) has been one of the most widely investigated proteins, presumably due to its central role in the common genetic disorder cystic fibrosis (CF). CFTR is localized in the apical plasma membrane of secretory epithelia mediating anion (Cl^-^ and HCO_3_^-^) efflux to the lumen. This protein consists of five domains: two transmembrane domains, two cytoplasmic nucleotide-binding domains (NBD) and a regulatory (R) domain [interested readers can find detailed reviews about the structure and the structure- function relationships of CFTR in recent publications (Liu et al., 2017; Callebaut et al., 2018)]. In non-stimulated cells the unphosphorylated R domain binds to NBD1 preventing the interaction of NBD1 with NBD2 and thus ATP hydrolysis, which is mandatory for the opening of the channel. During physiological stimulation agonist binding to adenylyl cyclase (AC) coupled receptors increases the intracellular cAMP and activate protein kinase A (PKA). Active PKA can phosphorylate the R domain resulting in its dissociation from NBD1 and allowing the interaction of NBD1 and NBD2. The released R domain forms connection between CFTR and other membrane proteins and transporters (Sheppard and Welsh, 1999; Guggino and Stanton, 2006a). One example of such a connection is the interaction of CFTR with the SLC26A6 Cl^-^/HCO_3_^-^ exchanger. The STAS domain of SLC26A6 interacts with the phosphorylated R domain of CFTR leading to the simultaneous activation of the two membrane proteins (Hong et al., 2014). Other CFTR domains mediate protein-protein interactions and stabilize CFTR in the apical membrane. These domains include postsynaptic density-95/disc-large/zonula occludens-1 (PDZ)-interacting domains, a protein phosphatase-2A-binding domain, AMP kinase-binding domain in the C terminus and syntaxin-1A-binding domain and synaptosome-associated protein binding domain in the N-terminus (Shenolikar et al., 2004; Brone and Eggermont, 2005; Guggino and Stanton, 2006a,b).

Cystic fibrosis transmembrane conductance regulator is found in several functionally different organs and tissues including lung, salivary glands, esophagus, stomach, biliary tract, sweat duct, intestine, kidney, heart, vas deferens and pancreas playing a fundamental role in the physiological secretory processes (Bradbury, 1999). Within the gastrointestinal tract CFTR expression varies among different tissues. In the gastric mucosa CFTR expression is lower (Strong et al., 1994) in contrast to the apical membrane of cholangiocytes (Strong et al., 1994), or small intercalated and proximal intralobular pancreatic ductal epithelial cells where high expression of CFTR can be detected (Marino et al., 1991). The levels of expression correlate with the significance of CFTR function. As an example CF is commonly complicated by exocrine pancreatic insufficiency suggesting the importance of CFTR function in pancreas physiology.

Cystic fibrosis transmembrane conductance regulator plays a fundamental role in the production of the alkaline, isotonic fluid secreted by pancreatic ductal cells in response to food intake that may contain up to 140 mM HCO_3_^-^. The physiological function of this alkaline secretion is to wash out the secreted digestive proenzymes from the pancreatic ductal tree into the duodenum and to neutralize acidic chyme entering the proximal part of the small intestine. Additionally, the alkaline fluid neutralizes the protons co-released during digestive enzyme secretion by pancreatic acinar cells (Behrendorff et al., 2010). However, a recent publication by Han et al. (2016) suggests that pancreatic acinar cells might be capable of HCO_3_^-^ secretion as well. Acinar cells express the Ca^2+^ activated Cl^-^ channel Anoctamin1 (ANO1) – but not CFTR – on the apical membrane, which channel’s anion selectivity can be dynamically modulated by the Ca^2+^/calmodulin complex making it permeable for HCO_3_^-^ (Jung et al., 2013). On the other hand the vital role of ductal secretion in the pancreatic physiology is supported by several observations. Impaired ductal secretion in CFTR knockout mice alters the membrane dynamics and endocytosis at the apical plasma membrane of pancreatic acinar cells (Freedman et al., 2001). In addition decreased ductal secretion in Na^+^/H^+^ exchanger regulatory factor-1 (NHERF1) knockout mice, due to the loss of CFTR leads to more severe experimental AP (Pallagi et al., 2014). More importantly pharmacological restoration of CFTR function in pancreatic ducts corrected acinar cell function and decreased pancreatic inflammation (Zeng et al., 2017). As the autoactivation of trypsinogen is a pH dependent process, which is accelerated in acidic pH ranges, the alkaline intraductal pH prevents the premature trypsinogen activation and thus protecting the pancreas from autodigestion (Pallagi et al., 2011).

**FIGURE 1:**
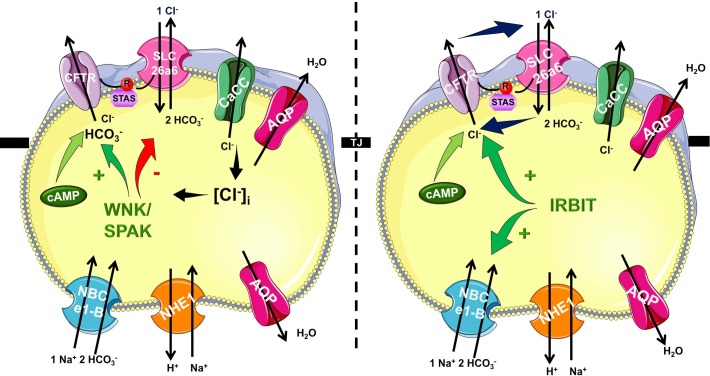
Mechanism of pancreatic ductal HCO_3_^-^ secretion. Pancreatic ductal cells secrete HCO_3_^-^ rich isotonic fluid as a result of a complex interplay by several transport proteins. HCO_3_^-^ is accumulated across the basolateral membrane Na^+^/HCO_3_^-^ cotransporter NBCe1-B. Via the luminal membrane HCO_3_^-^ is secreted by SLC26A6 and likely A3 Cl^-^/HCO_3_^-^ exchangers and cystic fibrosis transmembrane conductance regulator (CFTR) Cl^-^ channel. The operation of these transporters allows the pancreatic ductal cells to create 140 mM maximal bicarbonate concentration during stimulated secretion. The CFTR R domain and the STAS domain of the SLC26 Cl^-^/HCO_3_^-^ exchangers interact increasing the overall open probability of CFTR. At the beginning of the ductal system in the proximal ducts the intraluminal Cl^-^ concentration is high, and thus HCO_3_^-^ is secreted via Cl^-^/HCO_3_^-^ exchange regulated by IRBIT. In the distal part the intraluminal Cl^-^ drops and the low intracellular Cl^-^ concentration activates the WNK/SPAK kineses, which phosphorylate CFTR, switching the ion selectivity to HCO_3_^-^.

In the recent years it became very clear that HCO_3_^-^ efflux trough CFTR has a central role in the ductal secretion, which is the result of a strictly regulated interaction of the electrogenic SLC26A6 Cl^-^/HCO_3_^-^ exchanger and the CFTR Cl^-^ channel (Lee et al., 2012; Maleth and Hegyi, 2014) (Figure 1). Due to this interaction (between STAS and R domains, see above) the ductal cells are able to establish 140 mM maximal intraluminal HCO_3_^-^ concentration, which is ∼5–6 fold higher than the intracellular (Hong et al., 2014). The luminal Cl^-^ concentration is another factor in the coordination of CFTR permeability. According to the current model, in the proximal ducts the intraluminal Cl^-^ concentration is above 30 mM therefore in this case CFTR provides the extracellular substrate for the Cl^-^/HCO_3_^-^ exchange of SLC26A6. At the distal part of the pancreatic ductal tree the luminal and intracellular Cl^-^ concentration drops thus the anion exchange of SLC26A6 is insufficient. The low intracellular Cl^-^ activates the With-No-Lysine (WNK)/STE20/SPS1-related proline/alanine-rich kinase (SPAK) kinase pathway switching CFTR permeability in favor of HCO_3_^-^ (Park et al., 2010). The exact molecular mechanisms and regulation of pancreatic ductal secretion have been recently reviewed in details (Lee et al., 2012). Importantly, mutations in CFTR responsible for chronic pancreatitis (CP), but not CF were found to selectively modify the HCO_3_^-^ permeability of CFTR (LaRusch et al., 2014). The mutations identified in this study were associated with pancreatitis, but did not cause an ordinary CF phenotype. In a subsequent analysis, the authors concluded that these mutations cause the loss of WNK1/SPAK pathway-activated increase in the HCO_3_^-^ permeability of CFTR. Notably, since the Cl^-^ permeability is not affected, these mutations only damage the pancreas.

## Role of Ca^2+^ Signaling in the Regulation of Cftr

**FIGURE 2:**
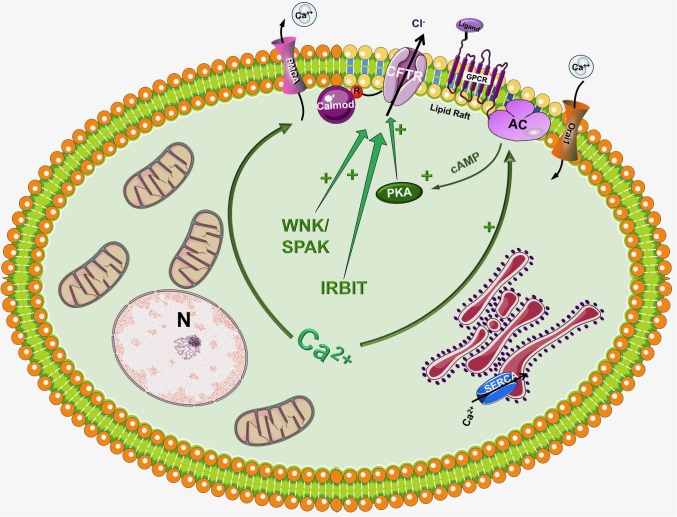
Regulation of CFTR by the intracellular Ca^2+^. Although CFTR is mainly considered as a cAMP regulated ion channel, several observations suggest that the intracellular Ca^2+^ can regulate CFTR activity by a complex mechanism. First of all, Ca^2+^ can increase the activity of several adenylyl cyclase (AC) isoform increasing the production of cAMP. In another suggested pathway Ca^2+^ activates CFTR, at least in part, through activation of the Pyk2/Src complex and tyrosine phosphorylation. Very recently Bozoky et al. showed that CFTR and calmodulin colocalize at the apical membrane of primary cells and that calmodulin interacts directly with the R domain of CFTR in a Ca^2+^- and phosphorylation-dependent manner. This interaction triggered an open probability of CFTR, which was similar to that due to PKA phosphorylation.

In the traditional view CFTR is a cAMP activated Cl^-^ channel as described above, however, in the recent years significant number of studies have highlighted the importance of Ca^2+^ signaling and the synergism between Ca^2+^ and cAMP signalization in the regulation of pancreatic epithelial functions (Ahuja et al., 2014) (Figure 2). Activation of CFTR by Ca^2+^ have been suggested earlier by Namkung et al. (2003). Using Capan-1, a pancreas adenocarcinoma cell line, which express endogenous CFTR, they showed that Ca^2+^ signals activate the Cl^-^ dependent HCO_3_^-^ transport of CFTR. This effect was completely absent in CFPAC-1 cells, which shows no CFTR expression, but adenovirus mediated overexpression of CFTR restored the stimulation. Using other models Seidler et al. (1997) found that in rodent small intestine a functional CFTR protein is required for cAMP-; cGMP and Ca^2+^ dependent HCO_3_^-^ secretion. They showed that 75–100% reduction of carbachol-stimulated HCO_3_^-^ secretion can be observed in Cftr knockout mice compared to wild type animals. Whereas inhibition of CFTR with CFTR_inh_-172 diminished the responses to cholinergic agonists in pig submucosal glands (Thiagarajah et al., 2004). In addition, pilocarpine, a Ca^2+^-mobilizing muscarinic agonist that is widely used in sweat Cl^-^ tests to measure CFTR activity, most likely affect CFTR through Ca^2+^ (Joo et al., 2010; Sun et al., 2010; Cho et al., 2011; Maleth et al., 2015a).

Another well-known fact is that the increase of intracellular Ca^2+^ influences the activity of different AC isoforms. The ACs inhibited by intracellular Ca^2+^/PKC/PKA are AC5 and AC6 while AC1, AC3 and AC8 are Ca^2+^/Calmodulin stimulated (Halls and Cooper, 2011; Willoughby, 2012). In a recent study Sabbatini et al. (2013) reported that AC3, AC4, AC6, AC7, and AC9 are expressed in the pancreatic ductal epithelia. The authors demonstrated that isolated ducts from AC6 knockout mice represented reduced cAMP generation, PKA activation and ductal fluid secretion. These results suggest that AC6 is important in the regulation of pancreatic fluid secretion in response to hormonal stimulation (such as secretin and VIP). The findings of this study raise interesting questions how synergy between Ca^2+^ and cAMP signaling is executed (since AC6 is inhibited by Ca^2+^). In a recent publication Park et al. (2013) suggested that inositol 1,4,5-triphosphate (InsP_3_) receptor-binding protein released with InsP_3_ (IRBIT) controls the synergy between the Ca^2+^ and cAMP signaling pathways. The authors demonstrated that co-expression of CFTR and SLC26A6 with IRBIT maximally activate the two proteins in response to low agonist concentration, whereas IRBIT knockdown reduced their activation. PKA activation with forskolin left shifted the concentration-dependence of SLC26A6 activation by Ca^2+^ without directly activating SLC26A6. In contrast, Ca^2+^ signaling evoked by carbachol enhanced activation of CFTR by cAMP. The synergy was mediated by intracellular release of IP_3_ rather than Ca^2+^ since the synergism by IRBIT was independent of cytoplasmic Ca^2+^.

Another interesting aspect of CFTR regulation is that different AC isoforms are localized in different nanodomains of the plasma membrane. The Ca^2+^ regulated AC1, AC5, AC6, and AC8 are targeted into lipid rafts in the plasma membrane, whereas the Ca^2+^ independent AC2 and AC7 are excluded from these rafts (Delint-Ramirez et al., 2011; Ahuja et al., 2014). This integrated subcellular localization could potentially promote the regulation of AC activity and thus intracellular cAMP levels through Ca^2+^. Supporting this idea in 2013 Namkung et al. (2010) have reported the regulation of CFTR by AC1. They suggested that CFTR in primary cultures of human bronchial epithelial cells is regulated by intracellular Ca^2+^ that activates AC1 and the downstream cAMP/PKA signaling. They have also found the colocalization of the two in the apical membrane of the cells suggesting that AC1-CFTR interaction is an important part of the cross-talk between the Ca^2+^ and cAMP signaling. A more recent study confirmed these finding by showing that α7 nAChR plays an important role in the regulation of CFTR function and in the pathogenesis of smoking-related chronic lung diseases (Maouche et al., 2013). The authors demonstrated that Ca^2+^ entry trough α7 nAChR activates AC1 and PKA, which in turn leads to CFTR activation. They found that α7 nAChR, CFTR, and AC1 are associated in supramolecular complexes within lipid rafts at the apical membrane of airway epithelial cells.

Billet et al. (2013) investigated in details how carbachol-induced elevation of cAMP increases CFTR activity. Using BHK cells that co-express CFTR and muscarinic acetylcholine receptor (M3R) they found that only ∼50% of the cholinergic response was evoked by Ca^2+^ induced activation of ACs; whereas after PKA inhibition, or removal of 15 PKA consensus sequences of CFTR, almost half of the CFTR response persisted. In further analysis the authors showed that Src Inhibitor-1 (Inh-1) (which inhibits Src tyrosine kinase) abolished the PKA-independent component of muscarinic stimulation. Based on their results the authors proposed that Ca^2+^ activates CFTR, at least in part, through activation of the Pyk2/Src complex and tyrosine phosphorylation. Another interesting aspect of PKA-independent activation of CFTR by Ca^2+^ elevation was recently reported by Bozoky et al. (2017). The authors showed that CFTR and calmodulin colocalize at the apical membrane of primary cells and that calmodulin interacts directly with the R domain of CFTR in a Ca^2+^-, and phosphorylation-dependent manner. This interaction triggered an open probability of CFTR, which was similar to that induced by PKA phosphorylation.

## Absence of Cftr Alters Ca^2+^ Homeostasis

**FIGURE 3:**
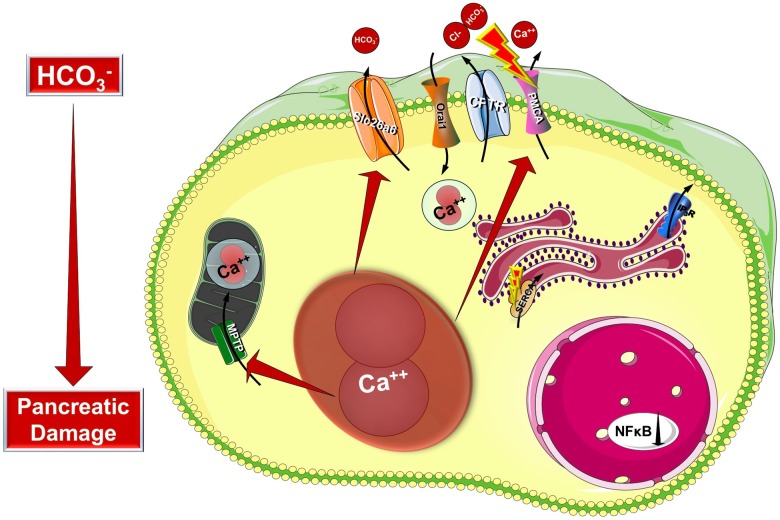
Intracellular changes in cystic fibrosis. Impaired expression of CFTR causes the condensation of the ER network due to the trapped CFTR protein in the ER lumen. This morphological change will result in IP_3_R clustering and increased IP_3_R activity complicated with increased SERCA, TRPC6, and Orai1 activity in the ER and plasma membrane, respectively. Importantly, the PMCA activity is impaired leading to sustained intracellular Ca^2+^ elevation. The intracellular Ca^2+^ overload promotes mitochondrial damage through increased mitochondrial Ca^2+^ uptake. These changes together will lead to further decrease of the epithelial ion secretion that could significantly damage the exocrine pancreas.

Cystic fibrosis transmembrane conductance regulator is a plasma membrane ion channel that extensively contributes to the epithelial ion and fluid secretion. Therefore it is not surprising that most research studies in CF focus on the effect of CFTR damage to the whole organ. However, CFTR has a complex integrative role in the intracellular signaling therefore it could be considered as a central signaling hub. Taken this into consideration we can presume that in cells with aberrant CFTR expression the intracellular signaling processes are influenced as well. Indeed, early studies in the 80s by Shapiro et al. highlighted that the intracellular Ca^2+^ concentration is elevated in different cell types (fibroblasts and lymphocytes) both from homozygous or heterozygous carriers as demonstrated by atomic absorption spectrophotometry (Feigal and Shapiro, 1979a; Shapiro and Lam, 1982, 1987). Moreover they demonstrated that the mitochondrial Ca^2+^ uptake is significantly increased in CF fibroblasts (Feigal and Shapiro, 1979b). By that time CFTR was not discovered and these observations suggested that CF might be caused by altered Ca^2+^ homeostasis and could be a mitochondria related disorder. This theory was eventually abolished by the identification of CFTR and reasonably, research interest was focused on its channel properties. However, the alterations of intracellular Ca^2+^ are still present, but shall be considered as consequences of the impaired CFTR function, or expression and not the cause of CF. More recent studies provided deeper understanding of the development of altered intracellular Ca^2+^ levels (Figure 3). As an underlying mechanism for altered Ca^2+^ signaling, increased IP_3_R-dependent Ca^2+^ response has been suggested in F508del-CFTR expressing airway epithelial cells, which was normalized following the correction of the abnormal trafficking of F508del-CFTR (Antigny et al., 2008b). Whereas Philippe et al. (2015) showed that both sarco/endoplasmic reticulum Ca^2+^-ATPase (SERCA) and plasma membrane Ca^2+^-ATPase (PMCA) pumps can contribute to the deregulation of the Ca^2+^ homeostasis in CF. The activity of the SERCA pump and the ER Ca^2+^ concentration were strongly increased, while PMCA function is significantly impaired in CF bronchial epithelial cells. They also confirmed the earlier findings on the elevated mitochondrial Ca^2+^ uptake in CF cells compared to controls. Treatment with the CFTR folding corrector VX-809 reversed the alterations of the intracellular Ca^2+^ signaling. Interestingly, they demonstrated the physical interaction of SERCA2b with CFTR, and PMCA with CFTR. Antigny et al. (2008a) have also found that once F508del CFTR was rescued by miglustat or low temperature in human CF-KM4 cells Ca^2+^ mobilization decreased compared to uncorrected cells. In another study the contribution of transient receptor potential canonical 6 (TRPC6) channel has been demonstrated to the increased intracellular Ca^2+^ levels. This study showed that CFTR and TRPC6 are functionally coupled within a molecular complex in airway epithelial cells, which is lost in CF leading to abnormally increased TRPC6-dependent Ca^2+^ influx (Antigny et al., 2011b). Balghi et al. (2011) found elevated extracellular Ca^2+^ entry in CFBE cells due to enhanced Orai1 insertion into the plasma membrane in CF leading to increased IL-8 secretion, which is a major contributor to the inflammation in CF. Thus, in the proposed model the F508del-CFTR mutation caused the condensation of the ER network due to the trapped CFTR protein in the ER. This morphological change will result in IP_3_R clustering and increased IP_3_R activity complicated with increased SERCA, TRPC6 and Orai1 activity in the ER and plasma membrane, respectively. In addition, the PMCA activity seems to be impaired (Antigny et al., 2011a). These together lead to increased ER Ca^2+^ concentration, which might further impair the folding of CFTR since decreasing and also maintaining low ER Ca^2+^ level promotes the correction of defective F508del-CFTR (Norez et al., 2006).

## Altered Mitochondrial Function in Cf

The intracellular Ca^2+^ signaling regulates mitochondrial function via mitochondrial Ca^2+^ uptake, which enhances the activity of tricarboxylic acid cycle dehydrogenases and the ATP synthase (Hansford and Zorov, 1998). Under physiological conditions the mitochondrial Ca^2+^ levels are tightly controlled by the mitochondrial Ca^2+^ uniporter (MCU) responsible for Ca^2+^ uptake (Baughman et al., 2011; De Stefani et al., 2011) and Na^+^/Ca^2+^ exchanger (NCLX) (Palty et al., 2010) for the efflux. Moreover in several cell types mitochondria act as Ca^2+^ buffers that help to prevent sustained global raises of intracellular Ca^2+^. However, sustained excess of cytosolic Ca^2+^, as seen in CF, can lead to mitochondrial Ca^2+^ overload and injury that ultimately activate apoptosis or necrosis. In addition, mitochondrial Ca^2+^ can induce the mitochondrial permeability transition pore (MPTP) opening that results in the loss of mitochondrial membrane potential, uncoupling of the respiratory chain and a consequent drop of mitochondrial ATP synthesis. The increased permeability of the inner mitochondrial membrane eventually leads to mitochondrial swelling, rupture, and necrotic cell death (Golstein and Kroemer, 2007; Halestrap, 2009). The investigation of mitochondrial dysfunction in CF – similarly to altered cytosolic Ca^2+^ handling – goes back to the 70s. In an early study Antonowicz et al. (1972) demonstrated an increased activity of the lysosomal α-glucosidase but did not find any defect in three of the mitochondrial enzymes tested (succinic dehydrogenase, glutamic dehydrogenase, and malic dehydrogenase). Few years later Feigal and Shapiro described increased mitochondrial Ca^2+^ uptake, mitochondrial oxygen consumption in mitochondria isolated from CF skin fibroblasts (Feigal and Shapiro, 1979b) and increased mitochondrial energy expenditure (Shapiro, 1988). Similarly, impaired Ca^2+^ handling, mitochondrial damage emerged as a consequence of CFTR mutations and not the cause of CF. More recently Antigny et al. (2009) showed that the mitochondrial network is fragmented in F508del-CFTR expressing airway epithelial cells, the ΔΨ_m_ is depolarized and Ca^2+^ uptake is reduced in CF mitochondria compared to control cells not effected by CF. They proposed that the deficient mitochondrial Ca^2+^ uptake is a consequence of mitochondrial membrane depolarization. Another study of F508del airway cells described impaired mitochondrial oxygen consumption, mitochondrial membrane potential, adenine nucleotide translocator-dependent ADP/ATP exchange and mitochondrial Complex I and IV activities, whereas both mitochondrial ROS production and membrane lipid peroxidation were increased (Atlante et al., 2016). The authors demonstrated that VX-809 treatment significantly improved all the parameters. On the other hand, the expression of MT-ND4, which is a mitochondrial gene encoding a subunit of the mitochondrial Complex I, seems to be CFTR-dependent. The gene expression is decreased if CFTR expression, or activity is impaired (Valdivieso et al., 2007), which in turn reduce the activity of mitochondrial Complex I (Valdivieso et al., 2012). As a consequence of these changes, Rimessi et al. (2015) demonstrated that the degree and quality of the inflammatory response in CF are supported by mitochondrial perturbation, which was dependent on the presence of *Pseudomonas aeruginosa* infection. In this process flagellin protein acted as the inducer of mitochondrial dysfunction leading to MCU mediated mitochondrial Ca^2+^ overload, which in turn leads to inflammasome NLRP3 activation, IL-1β and IL-18 processing (Rimessi et al., 2015). In accord with the mitochondrial dysfunction, several work described increased apoptotic cell death in CF (Valdivieso and Santa-Coloma, 2013). As the underlying mechanism some studies suggest that the increased apoptosis is a result of recurrent bacterial infections (Jendrossek et al., 2003; Kirschnek and Gulbins, 2006), while others proposed that it is a primary consequence of CF that occurs without infection (I’Hoste et al., 2010; Rottner et al., 2011). The alterations include enhanced ROS production, release of cytochrome c mitochondrial depolarization and also the activation of c-Jun N-terminal kinases (JNKs) (Jendrossek et al., 2003). Other studies highlight that increased apoptosis might be due to the impaired oxidant-antioxidant balance, which may contribute to the inflammation in CF [reviewed recently in I’Hoste et al. (2010), Rottner et al. (2011), and Ziady and Hansen (2014)]. Rottner et al. (2007) have also linked the NF-κB pathway to the initiation of apoptosis.

## The Role of Cftr in Pancreatic Disease Pathogenesis

### Cystic Fibrosis

Cystic fibrosis is the most common autosomal recessive genetic disease characterized by multiorgan pathology and significantly decreased life expectancy caused by the impaired function or expression of CFTR. Due to recent therapeutic developments, the life expectancy of patients with CF elevated significantly in the past decades, however, the average age of death (usually caused by respiratory failure) is still 31.4 years (Annalisa Orenti et al., 2016). In addition, 31% of the CF patients have a chronic lung infection caused by *P. aeruginosa*, whereas 83% of all CF patients are in need of pancreatic enzyme replacement therapy causing a significant burden to the patients and to the healthcare systems.

Currently more than 2000 *CFTR* gene mutations have been described, whereas only 159 mutations have been characterized in terms of disease liability (Sosnay et al., 2013; De Boeck and Amaral, 2016; Bolia et al., 2018). The most common mutation type in 85% of patients worldwide is the deletion of phenylalanine at position 508 (F508del), which was identified by Riordan et al. (1989). To date mutations are classified into seven different groups according to the CFTR defect caused (De Boeck and Amaral, 2016; Hegyi et al., 2016): Class I mutations, which include frame shift, splicing or non-sense mutations that introduce premature termination codons; Class II mutations, which lead to misfolding and impaired protein biogenesis at the ER; Class V mutations which result in reduced synthesis due to promoter or splicing abnormalities; and Class VI mutations that destabilize the channel in post-ER compartments and/or at the PM. Whereas Class III and IV mutations impair the gating and channel pore conductance, respectively, thus selectively compromising CFTR function. In case of Class VII mutations no messenger RNA can be detected. The disease affects all CFTR expressing epithelia, but pancreas is one of the organs that’s harmed the earliest and damaged most severely by CF (Wilschanski and Novak, 2013). Apparently the lack of CFTR has a dramatic impact on the exocrine pancreatic secretion due to its central role in this process (see above). Pancreatic damage results in severe inflammation, secretion of viscous, protein rich fluid leading to obstruction of ducts by mucoprotein plugs, calcification, the destruction of acini, pancreatic cyst formation and lastly, generalized fibrosis. The changes begin in utero and eventually will lead to pancreatic insufficiency (PI), which is manifested in 83% of all CF patients. Clinical symptoms of PI are abdominal pain, maldigestion and lowered BMI. Interestingly, 15% of the patients have evidence of pancreatic damage, but retain sufficient endogenous exocrine pancreatic function to sustain normal digestion (PS). This subgroup of patients is at an increased risk of developing AP. In an elegant study by Ooi et al. (2011) investigated the association between severity of CFTR genotype and the risk of developing AP. The involved 277 PS patients were divided into three groups (severe, moderate-severe and mild) based on the severity of pancreatic insufficiency. They showed that patients in the mild group have an increased risk of pancreatitis compared to moderate-severe and severe groups.

### Acute Pancreatitis

**FIGURE 4:**
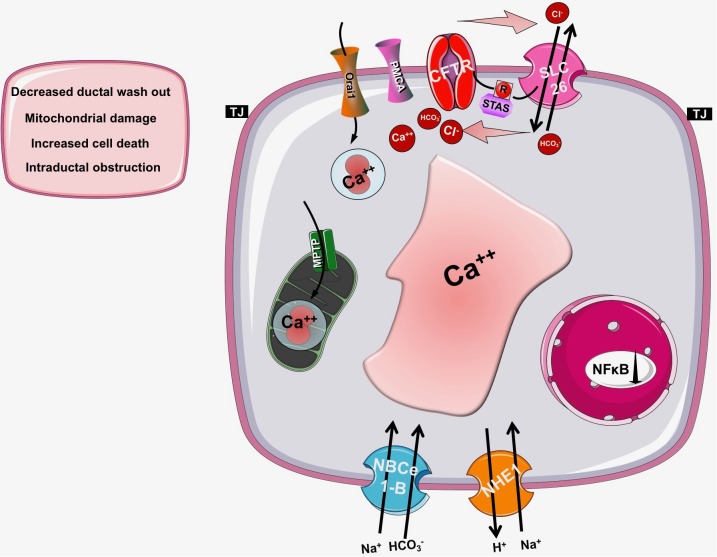
The role of CFTR in pancreatic diseases. In cystic fibrosis, acute or chronic pancreatitis the function of CFTR is impaired and/or its expression is decreased leading to insufficient bicarbonate and fluid secretion and a consequent drop in the intraluminal pH. These changes will decrease the washout of the digestive enzymes and increase the possibility of mucoprotein plug formation within the pancreatic duct lumen, which further decreases the pancreatic secretion. These observations suggest that the pancreatic ductal HCO_3_^-^ and fluid secretion and specifically CFTR channel have a fundamental role in the pathogenesis of pancreatic diseases.

As detailed above, CFTR has a central role in the pancreatic ductal secretion, whereas impaired function and/or expression of CFTR in CF leads to exorine pancreatic damage. Taken these into consideration, it seems reasonable to assume, that CFTR might have a potential role in the pathogenesis of AP as well (Hegyi et al., 2016) (Figure 4). Indeed, several previous studies suggested this relationship. Dimagno et al. found that the impaired CFTR expression in general knockout or in F508del mice caused overexpression of proinflammatory cytokines and increased the severity of cerulein-induced AP (DiMagno et al., 2005; DiMagno et al., 2010). Recently, our group analyzed the role of CFTR in the pathogenesis of alcohol-induced AP. *In vivo* and *in vitro* pancreatic fluid and HCO_3_^-^ secretion were already decreased in Cftr knockout mice, which impaired further after treatment with ethanol and fatty acids (Maleth et al., 2015a). In addition, Cftr knockout mice developed more severe AP upon ethanol and fatty acid treatment. The detailed analysis of the underlying mechanism highlighted that ethanol and fatty acids dose-dependently reduced the activity and expression of CFTR in pancreatic ductal cells leading to inhibited epithelial secretion. On the other hand, ethanol and fatty acids decreased the expression and plasma membrane density of CFTR due to accelerated protein turnover at the apical membrane and the protein folding in the endoplasmic reticulum was damaged as well. The activity of CFTR was inhibited by sustained elevation of intracellular Ca^2+^, decrease in cAMP, depolarization of mitochondrial membranes, and depletion of ATP caused by ethanol and fatty acids. The pivotal role of mitochondrial damage in this process was further highlighted by Judak et al. (2013). The authors demonstrated that ATP supplementation restored CFTR activity in pancreatic ductal cells treated with ethanol and fatty acids.

The other most common form of the disease is biliary AP, which is caused by choledocholithiasis complicated with outflow obstruction due to impacted gallstones. Pancreatic ductal secretion is likely reduced in patients with biliary AP, as suggested by the decreased intraductal pH, which correlates with the severity of AP (Takacs et al., 2013). Under experimental conditions, low concentration of chenodeoxycholate (CDCA) stimulated, in contrast high concentrations of CDCA strongly inhibited pancreatic ductal HCO_3_^-^ secretion by causing sustained Ca^2+^ elevation and severe mitochondrial damage (Venglovecz et al., 2008; Maleth et al., 2011). Although direct measurements are missing yet, it is plausible that the consequent intracellular ATP depletion will affect ATP-dependent transporters like CFTR.

### Chronic Pancreatitis

Chronic pancreatitis (CP) is the progressive inflammation of the pancreas accompanied by irreversible functional (exocrine and endocrine pancreatic insufficiency) and structural changes (ductal dilatation, irregulation, and calcification) (Tattersall et al., 2008). The incidence of the disease varies between 5 to 12/100,000 (Yadav and Lowenfels, 2013). Moreover on the vast majority of cases patients suffer from abdominal pain which significantly decreases their quality of life (Gardner et al., 2010). Current treatment is limited to enzyme replacement, pain relief and surgical interventions (pancreas head resection). Ko et al. (2010) showed earlier that the HCO_3_^-^ concentration in the pancreatic juice is decreased in CP (Braganza et al., 2011), in accord trafficking of CFTR is largely compromised and the protein is retained at the cytoplasm of pancreatic ductal cells (Ko et al., 2010). On the other hand Balazs et al. (2018) recently demonstrated that epithelial fluid secretion defect together with mucus hypersecretion lead to mucin accumulation in the small ducts, which can lead to ductal obstruction in CP. These alterations can reduce intraluminal pH, decrease the washout of the digestive enzymes and mucoprotein plug formation within the pancreatic duct lumen (Ko et al., 2012), which further decreases the pancreatic secretion. These observations suggest that the pancreatic ductal HCO_3_^-^ and fluid secretion and specifically CFTR channel have a fundamental role in the pathogenesis of CP.

## Closing Remarks

In this review we emphasize the effect of CFTR on intracellular signaling events focusing on the intracellular Ca^2+^ signaling and mitochondrial dysfunction. As detailed above both impaired regulation of the intracellular Ca^2+^ concentration and the mitochondrial damage develop due to complex mechanisms that can further ameliorate cell function and increase cellular damage complicating the organ damage in CF. These alterations have been described mainly in airway epithelial cells and we have very limited information how these mechanisms contribute to CF-related cell damage in other organs. However, taken into consideration that CFTR is highly expressed in the polarized pancreatic ductal cells and plays a pivotal role in the exocrine cell secretion, we could assume that these intracellular changes have a central role in the development of exocrine pancreatic damage in CF. In a recent case report Kounis et al. (2018) demonstrated that Ivacaftor treatment (a clinically used potentiator of CFTR) significantly improved the fecal elastase-1 level and decrease the number of pancreatic bouts per month in a patient with heterozygous mutation class III CFTR gene mutation. This is the first report suggesting that restoration of CFTR activity might improve exocrine pancreatic function in CF patients. It would be interesting to test whether the altered intracellular signaling mechanisms might be reversed as well. In addition, impaired CFTR function and expression were observed in AP as well leading to more severe pancreatic damage. Since toxic intracellular Ca^2+^ elevation and mitochondrial damage are hallmarks of AP, restoration of CFTR activity, or expression could potentially decrease the cell damage in AP (Mukherjee et al., 2008; Maleth et al., 2013, 2015b; Maleth and Hegyi, 2016). Further studies are needed to characterize these pathogenic steps in the exocrine pancreas, which could lead to better understanding of the disease pathogenesis and identification of potential targets that could be used to treat pancreatic insufficiency in CF.

## Author Contributions

TM and PP collected, analyzed, and summarized the literature and drafted the paper. JM collected and analyzed the literature and drafted the manuscript.

## Conflict of Interest Statement

The authors declare that the research was conducted in the absence of any commercial or financial relationships that could be construed as a potential conflict of interest.
